# Brown adipose tissue-derived exosomes mitigate the metabolic syndrome in high fat diet mice

**DOI:** 10.7150/thno.43968

**Published:** 2020-07-09

**Authors:** Xueying Zhou, Zhelong Li, Meihao Qi, Ping Zhao, Yunyou Duan, Guodong Yang, Lijun Yuan

**Affiliations:** 1 Department of Ultrasound Diagnostics, Tangdu Hospital, Fourth Military Medical University, Xi'an, People's Republic of China.; 2 The State Laboratory of Cancer Biology, Department of Biochemistry and Molecular Biology, Fourth Military Medical University, Xi'an, People's Republic of China.; 3 Department of Otolaryngology Head and Neck Surgery, Xijing Hospital, Fourth Military Medical University, Xi'an, People's Republic of China.

**Keywords:** Exosomes, brown adipose tissue, obesity, cell-free therapy, cardiovascular diseases

## Abstract

The ever-increasing incidence of obesity and related disorders impose serious challenges on public health worldwide. Brown adipose tissue (BAT) has strong capacity for promoting energy expenditure and has shown great potential in treating obesity. Exosomes are nanovesicles that share the characteristics of their donor cells. Whether BAT derived exosomes (BAT-Exos) might exert similar metabolic benefits on obesity is worthy of investigation.

**Methods:** Obese mice were established by high-fat-diet (HFD) feeding and were treated with Seum-Exos or BAT-Exos isolated from young healthy mice. Blood glucose, glucose tolerance and blood lipids were tested in mice with indicated treatments. Histology examinations were performed on adipose tissue, liver and heart by HE staining and/or Oil Red O staining. Echocardiography was performed to evaluate cardiac function of mice. *In vivo* distribution of exosomes was analyzed by fluorescence labeling and imaging and *in vitro* effects of exosomes were evaluated by cell metabolism analysis. Protein contents of BAT-Exos were analyzed by mass spectrometry.

**Results:** The results showed that BAT-Exos reduced the body weight, lowered blood glucose and alleviated lipid accumulation in HFD mice independently of food intake. Echocardiography revealed that the abnormal cardiac functions of HFD mice were significantly restored after treatment with BAT-Exos. Cell metabolism analysis showed that treatment with BAT-Exos significantly promoted oxygen consumption in recipient cells. Protein profiling of exosomes demonstrated that BAT-Exos were rich in mitochondria components and involved in catalytic processes.

**Conclusions:** Collectively, our study showed that BAT-Exos significantly mitigated the metabolic syndrome in HFD mice. Detailed elucidation of the reactive molecules and mechanism of action would provide new insights in combating obesity and related disorders.

## Introduction

Obesity refers to the state in which body fat accumulates to a degree that is harmful to health. At present, obesity is generally defined as having a body mass index (BMI) higher than 30 kg/m^2^ 1. With the spread of nutrient excess and sedentary lifestyle, the incidence of obesity keeps rising and it has now become a serious health burden worldwide. The excessive accumulation of adipose tissue causes several adverse effects on human health. It has been widely acknowledged that obesity is closely related with multiple diseases such as cardiovascular diseases 2-4, non-alcoholic fatty liver disease (NAHLD) 5, nonalcoholic steatohepatitis (NASH) 6, diabetes 7, 8, atherosclerosis 9, 10, neurological disorders 11, 12 and cancer 13-15. Addressing the challenges posed by obesity is now imperative.

Obesity is closely related with adipose tissue. There are two distinct kinds of adipose tissues in mammals, white adipose tissue (WAT) which stores energy and brown adipose tissue (BAT) which burns energy for heat. BAT mainly exists in infants and helps the babies to maintain their body temperatures by dissipating chemical energy as heat. BAT is rich in mitochondria and has high oxygen consumption rate. The fact that BAT can promote energy consumption has rendered it an ideal target for treating obesity and related disorders. Numerous researchers have focused on utilizing metabolically active BAT to treat obesity and the results are encouraging 16. Many studies have shown that transplantation of BAT in obese mice can lead to reduced body weight, lowered blood glucose and improved insulin sensitivity. The mechanisms for the beneficial effects of BAT transplantation involve enhanced endogenous BAT activity 17, increased energy expenditure and secretion of BAT adipokines including adiponectin 18, IL-6 19, and ANGPTL4 20. Despite the therapeutic effects and metabolic benefits, BAT transplantation can hardly be applied to clinical translation. The first and foremost challenge is the acquisition of the donor tissue. Immune intolerance is another issue that should be addressed prudently. Therefore, we hoped to find a way to preserve the metabolic benefits of BAT without tissue transplantation.

The secretome of BAT accounts for an important part of its metabolic influences 21, 22. In reviewing the secretory factors of BAT, we focused our research scope on exosomes. Exosomes are nanoscale lipid bilayer vesicles that contain various bioactive molecules. The bioactive molecules contained in the exosomes can interact with the recipient cells, leading to physiological and pathological changes of the recipient cells. There has been growing acknowledgement that exosomes play vital roles in intercellular communication 23, 24. Exosomes are released by cells, so the payloads of exosomes are often cell-specific. In another word, exosomes often share the properties of the donor cells. Besides, exosomes have several advantages over cell therapy including easy manipulation, high biological penetration, and immune tolerability and has been considered as superior candidate of cell-free therapy 25, 26. Therefore, it is reasonable to infer that BAT exosomes (BAT-Exos) might exert metabolic benefits on obese mice as BAT does which would render a promising strategy to combat obesity.

In the present study, we explored the metabolic benefits of BAT-Exos in treating obesity and the underlying mechanisms. The results showed that BAT-Exos effectively mitigated the metabolic syndromes of obese mice including high blood glucose, hepatic lipid accumulation and cardiac dysfunction. Then we demonstrated that BAT-Exos exerted metabolic effects at least partially by targeting liver and promoting energy expenditure. Mass spectrometry revealed that BAT-Exos function possibly via conferring components related with metabolism. Our study not only confirms the benefits of BAT-Exos, but also sheds light on engineering exosomes similar as BAT-Exos.

## Results

### Metabolic disorder and cardiac dysfunction in obese mice induced by high-fat-diet feeding

Mice were randomly allocated to high-fat diet (HFD) for induction of obesity or normal chow diet (NCD) to serve as lean controls. The differences of metabolism and cardiac function between obese mice and lean mice were then compared. After 16 weeks of feeding, mice in HFD group obtained significantly greater body weights and higher blood glucose levels, compared to mice in NCD group (Figure S1A-S1C). Histology examination by HE staining showed remarkable liver steatosis in HFD mice (Figure S1D). Evaluation of cardiac function by echocardiography showed reduced ejection fraction (EF) and fractional shortening (FS) in HFD mice compared to NCD mice, suggesting decreased systolic function of obese mice (Figure S1E and S1F). As for diastolic function, HFD mice exhibited abnormal diastolic function as revealed by reversed E'/A' ratio via tissue Doppler of mitral annulus (Figure S1G-S1J). Together, the above results demonstrated metabolic disorder and cardiac dysfunction in obese mice.

### BAT-Exos improve metabolism and restore hepatic and cardiac function in HFD mice

To study the effects of BAT-Exos on obese mice, exosomes were thus isolated from the interscapular brown adipose tissue in young healthy mice and characterized via exosome identification methods, including particle size distribution, transmission electronic microscopy and western blotting (Figure S2). About 30 µg -50 µg exosomes in protein concentration could be obtained from the BAT per mouse.

**Figure 1:**
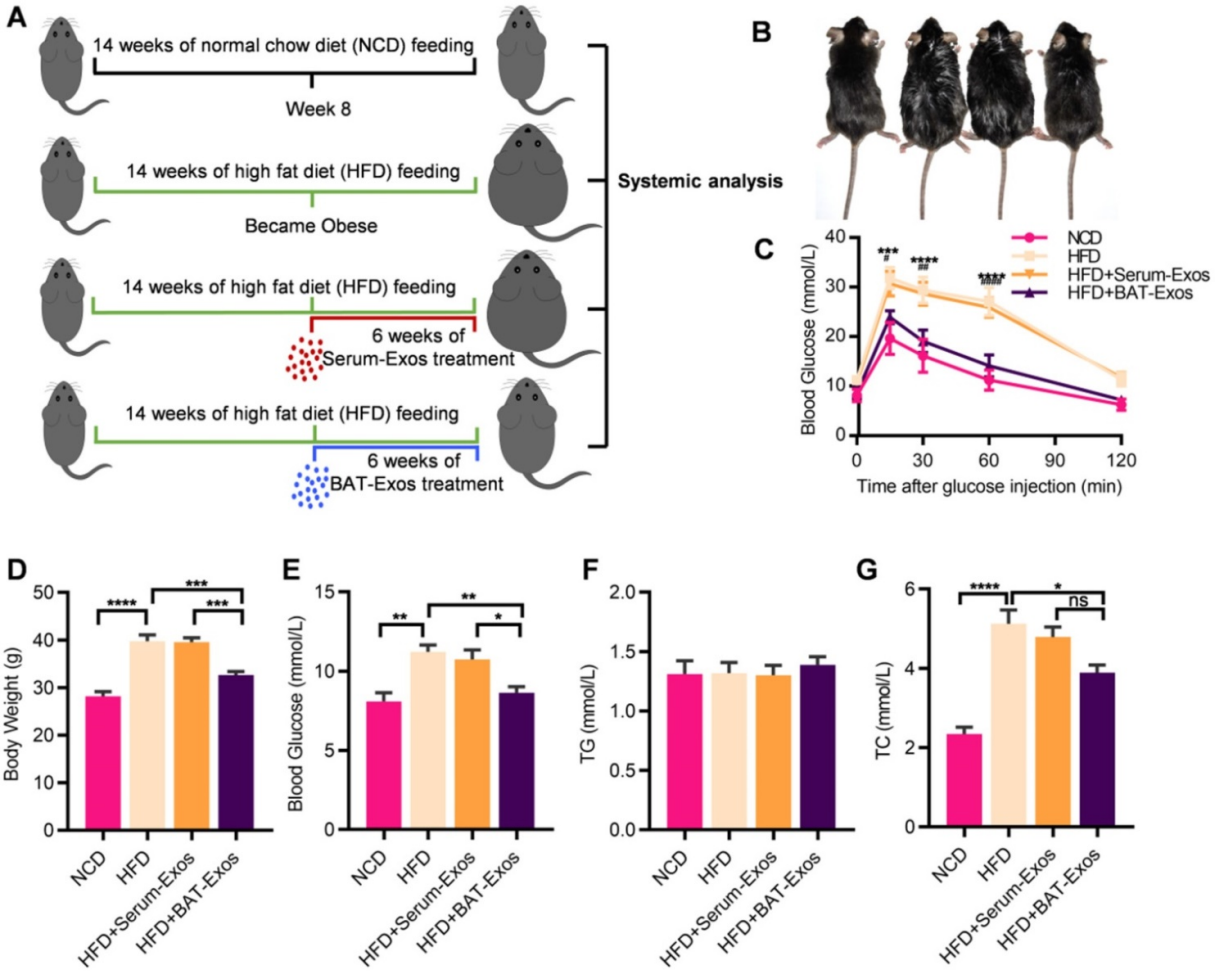
** BAT-Exos alleviate metabolic disorders in HFD mice. A.** Illustration of animal grouping and experimental procedures. Mice were allocated to either normal chow diet or high fat diet **B.** Representative images of mice from each group. **C.** Blood glucose during the intraperitoneal glucose tolerance test. ^*^ indicates significance between NCD versus HFD; ^#^ indicates significance between HFD versus HFD+BAT-Exos. **D-E.** Body weight (D) and blood glucose (E) of indicated mice. **F-G.** Serum lipid levels among different groups. Data are presented as mean ± SEM. n=6 per group, ^*^P < 0.05, ^**^P < 0.01, ^***^P < 0.001, ^****^P < 0.0001. HFD, high-fat diet; NCD, normal chow diet; TC, total cholesterol; TG, triglyceride.

In animal experiments, 6 weeks old male C57/BJ6 mice were divided into four groups: NCD, HFD, HFD plus treatment with Serum-Exos, HFD plus treatment with BAT-Exos. Detailed grouping and experimental design were illustrated in Figure 1A. Due to the shaving before ultrasound imaging, the body weight of mice dropped 1-2g on average. Treatment with BAT-Exos ameliorated the body weight gain and blood glucose elevation of HFD mice (Figure 1D and 1E), while there was no significant difference of food uptake (Figure S3). Glucose tolerance test via intraperitoneal injection demonstrated impaired glucose tolerance in HFD mice and treatment with BAT-Exos improved glucose tolerance in HFD mice (Figure 1C). As for blood lipid tests, there was no significant difference in triglycerides among different groups. Compared with mice fed normal diet, mice fed high-fat diet had higher cholesterol and the cholesterol level decreased only after BAT-Exos treatment (Figure 1F-1G). The control serum-Exos showed no obvious effects on the above metabolic parameters. To avoid potential confusion for control exosomes, we also tested the differences of the metabolic effects between serum and plasma exosomes. The results showed that either Serum-Exos or Plasma-Exos have no obvious effects on the metabolic parameters (Figure S4).

We further tested the expression of inflammatory cytokines in the liver and visceral adipose tissue (VAT). The decreased expression of the two inflammatory genes (TNFα and IL1β) in liver and VAT by BAT-Exo was consistent with the reduced fatty liver and improved metabolism (Figure S5). However, no differences in the white blood cell population were found (Table S1).

**Figure 2:**
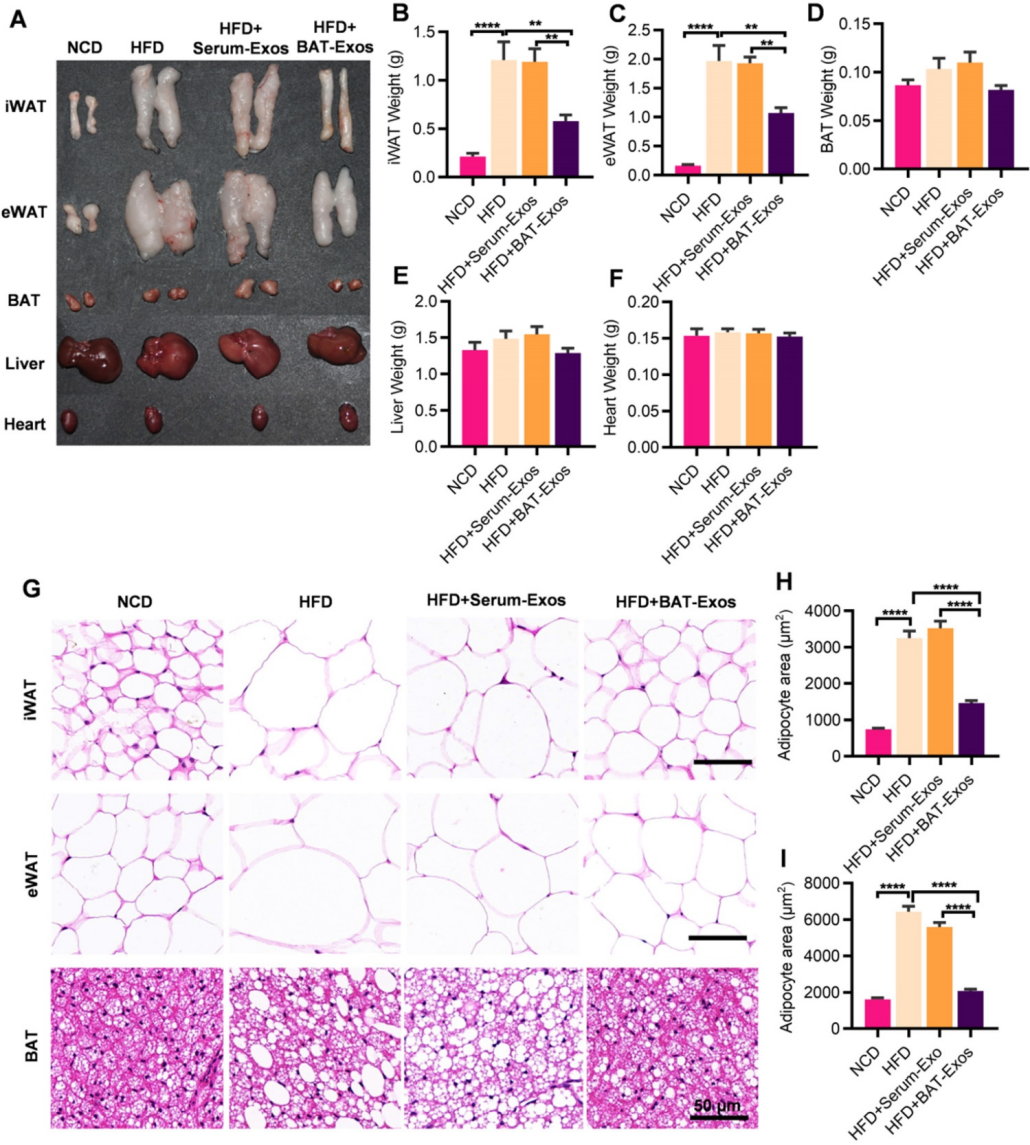
** BAT-Exos reduce white adipose tissue deposition and sizes of adipocytes. A.** Representative images of tissues harvested from indicated mice. **B-F.** Weights of different tissues of indicated mice. **G.** HE staining of iWAT (top), eWAT (middle) and BAT (bottom) from indicated mice. **H-I.** Quantification of adipocyte areas of iWAT (H) and eWAT (I). Data are presented as mean ± SEM. n=6 per group, ^**^P < 0.01, ^****^P < 0.0001. BAT, brown adipose tissue; eWAT, epididymal white adipose tissue; iWAT, inguinal white adipose tissue. Scale bar: 50 µm.

Mice were then sacrificed and major metabolic organs including heart, liver, BAT, iWAT (inguinal white adipose tissue) and eWAT (epididymal white adipose tissue) were harvested for further analyses (Figure 2A). According to the results, high-fat-diet feeding led to increased fat deposition in mice. Significant decreases in WAT weights were observed in BAT-Exos-treated HFD mice. No obvious changes in the weights of liver, heart and BAT among different groups were observed (Figure 2B-2F). Histology examination of WAT revealed prominent decrease of adipocyte size after BAT-Exos treatment. Lipid accumulation in BAT also reduced in HFD mice treated with BAT-Exos (Figure 2G-2I).

**Figure 3:**
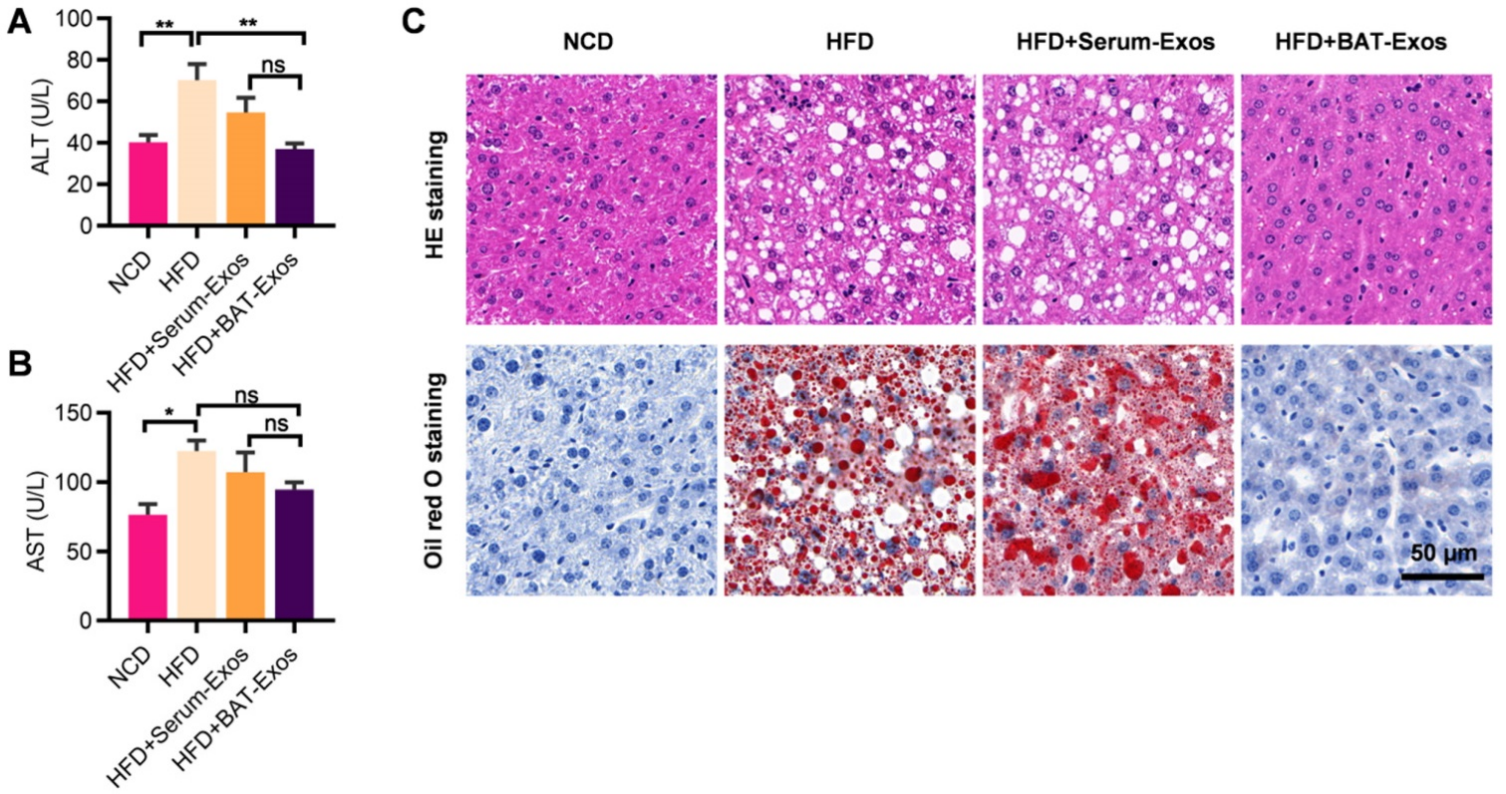
** BAT-Exos improve hepatic function and alleviate fatty liver. A-B.** Serum levels of ALT (A) and AST (B) in mice from each group. **C.** HE staining (top) and Oil Red O staining (bottom) of liver section of mice with indicated treatments. Data are presented as mean ± SEM. n=6 per group, ^*^P < 0.05, ^**^P < 0.01. ALT, alanine aminotransferase; AST, aspartate aminotransferase.

As liver and heart are closely associated with obesity-related adverse effects, we next explored the influences of BAT-Exos on the liver and heart of HFD mice. Serum ALT and AST were examined for evaluation of haptic function. The results showed that serum ALT and AST levels increased in HFD mice compared to NCD mice, suggesting impaired liver function in HFD mice (Figure 3A and 3B). Treatment with BAT-Exos lowered ALT and AST to a level similar in NCD mice. Histology analysis of liver by HE staining and Oil Red O staining revealed obvious steatosis in the liver of HFD mice with no treatment or HFD mice treated with Serum-Exos, while minimal lipid deposition in liver was detected in HFD mice treated with BAT-Exos (Figure 3C). These data suggested that BAT-Exos could reduce fat deposition in liver and improve hepatic function.

**Figure 4:**
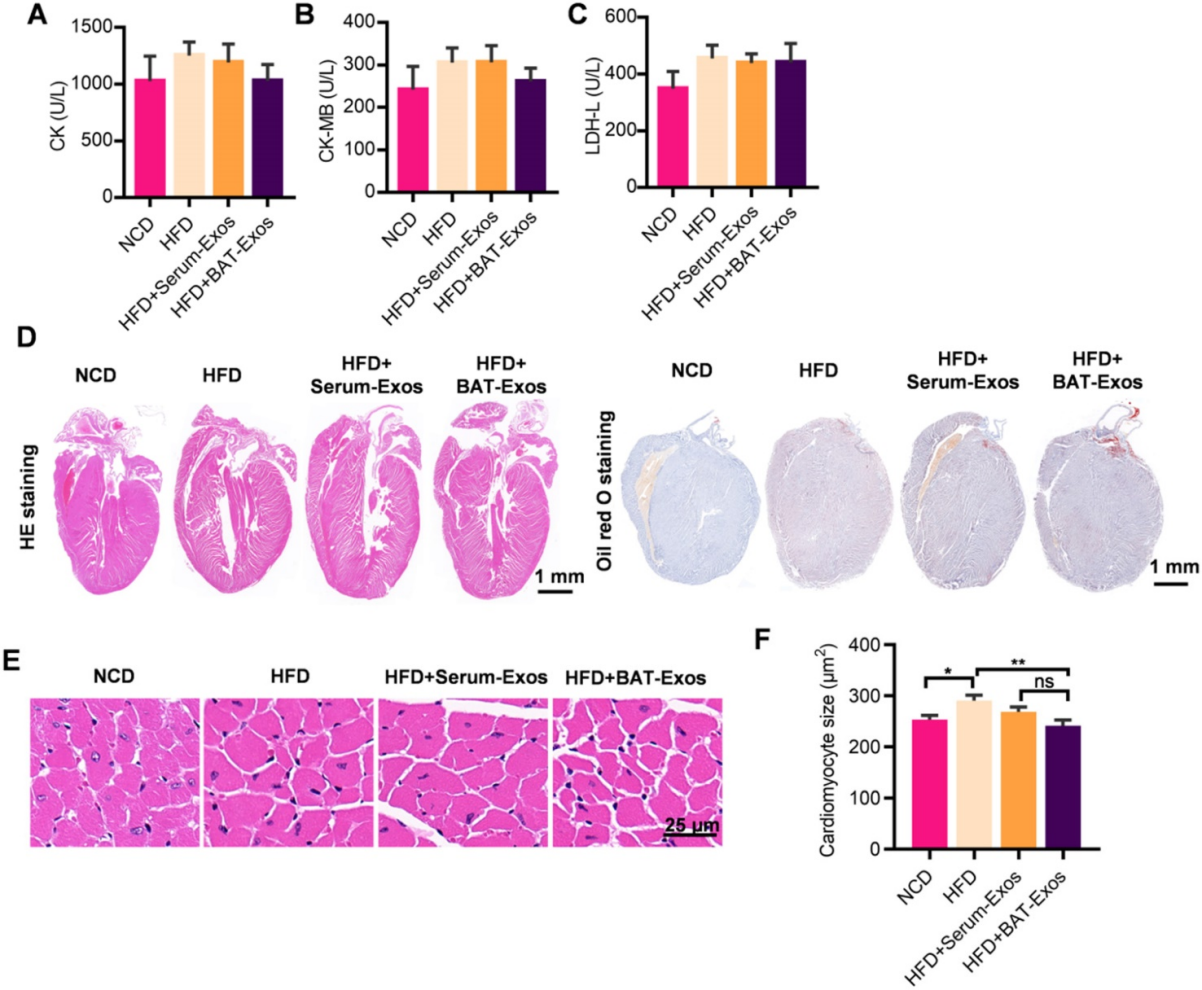
** BAT-Exos reduce cardiomyocyte hypertrophy in HFD mice. A-C.** Myocardial enzyme levels of indicated mice. A tendency towards higher myocardial enzyme level in HFD mice compared to BAT-Exos-treated mice were seen with no statistical significance. **D.** Histology analysis of heart of indicated mice by HE staining (left) and Oil Red O staining (right). **E.** Magnification of representative images of HE staining of heart. **F.** Quantitative analysis of cardiomyocyte sizes of mice with indicated treatment. Data are presented as mean ± SEM. n=6 per group, ^*^P < 0.05, ^**^P < 0.01.

**Figure 5:**
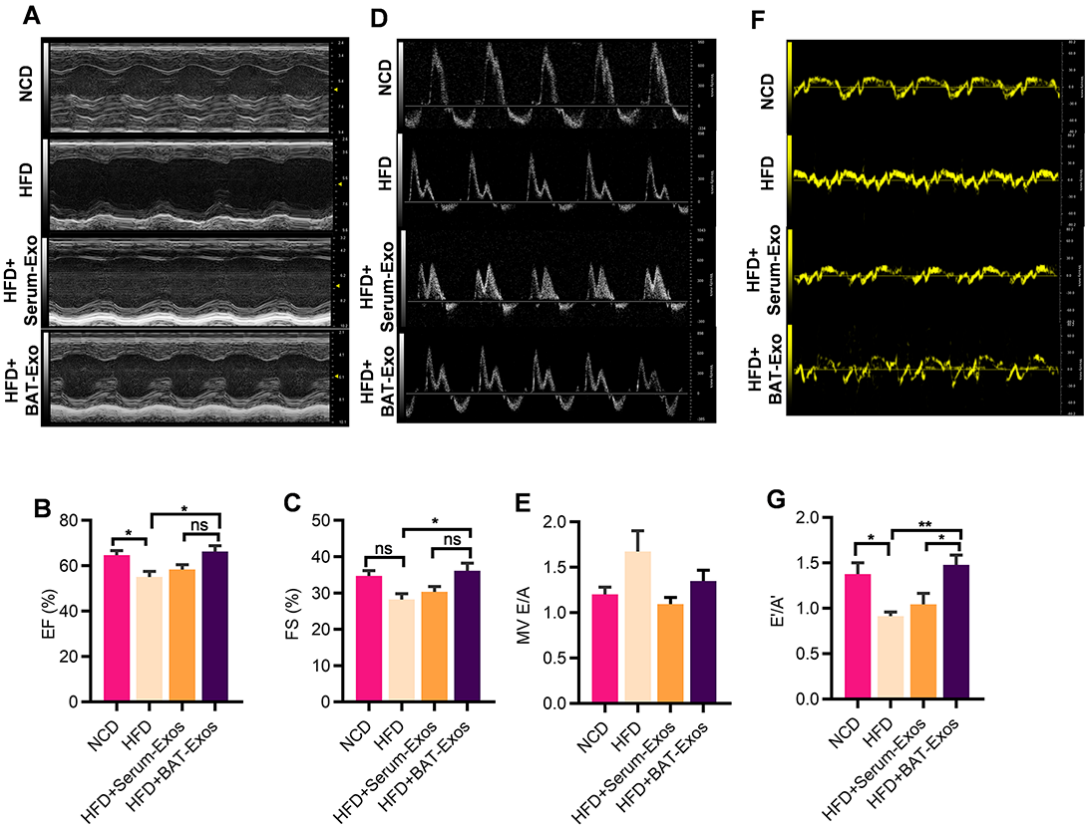
** BAT-Exos restore cardiac function in HFD mice. A.** M mode echocardiography of mice with different treatment for evaluation of systolic function. **B-C.** Quantification of systolic function parameters EF (B) and FS (C). **D.** Mitral flow measured by Doppler echocardiography. **E.** Quantification of diastolic function parameter E/A. **F.** Tissue Doppler of mitral annulus for evaluation of diastolic function of mice. **G.** Quantification of diastolic functional parameter E'/A'. Data are presented as mean ± SEM. n=6 per group. ^*^P < 0.05, ^**^P < 0.01. EF, ejection fraction; FS, fractional shortening; MV, mitral valve.

As regard to evaluation of cardiac structure and function, echocardiography, serum myocardial enzyme level tests as well as histology analysis by HE staining were performed. As shown in figure 4A to 4C, there were tendencies of higher myocardial enzyme level in HFD mice and reduced myocardial enzyme level in BAT-Exos-treated HFD mice, but the differences were not statistically significant (Figure 4A-4C). After magnification of the results of HE staining, enlargement of cardiomyocyte was seen in HFD mice and the average areas of cardiomyocytes decreased in BAT-Exos-treated HFD mice (Figure 4D-4F). The results of echocardiography examination showed that HFD mice exhibited reduced ejection fraction and fractional shortening and that treatment with BAT-Exos improved the impaired systolic function in HFD mice (Figure 5A-5C). Tissue doppler of mitral annulus showed that HFD mice exhibited reversed E'/A' ratio and that E'/A' ratio restored after BAT-Exos treatment, suggesting improved cardiac function in HFD mice after treatment with BAT-Exos (Figure 5F and 5G). Altogether, the above data clearly demonstrated the beneficial effects of BAT-Exos on cardiac function in obese mice.

We further evaluated whether BAT-Exos regulate the metabolism in a dose-dependent manner by injecting different doses of BAT-Exos. Only some of the metabolic parameters showed improvement in mice received 50 µg BAT-Exos, but more robust changes were seen in mice treated with higher doses of BAT-Exos (Figure S6), further confirming that BAT-Exos worked in dose-dependent manner.

### BAT-Exos preferentially target liver after intravenous injection and promote energy expenditure in hepatocytes

**Figure 6:**
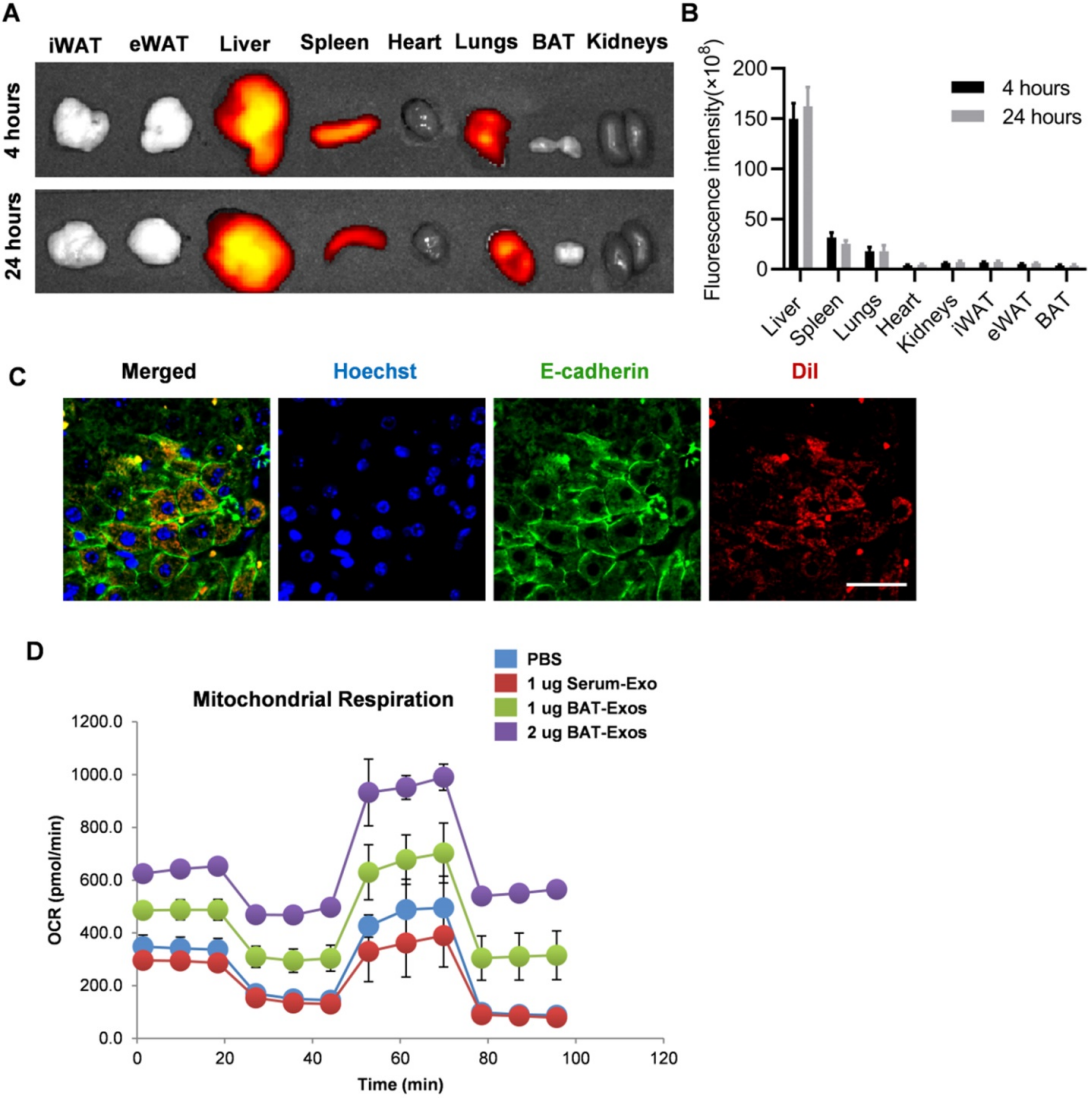
** BAT-Exos preferentially accumulate in liver and promote oxygen consumption in hepatocytes. A.** Representative images of *in vivo* distribution of DiR-labeled exosome by *in vivo* imaging system. Exosomes were labeled with fluorescent dye DiR and injected into mice via intravenous injection. **B.** Quantification of fluorescence intensity of different organs. **C.** Confocal microscopy of liver section. Colocalization of DiI-labeled exosomes with E-cadherin. Scale bar=25 µm. **D.** Cell oxygen consumption rate (OCR) of AML12 cells with indicated treatment measured by seahorse cell metabolism analyzer. eWAT, epididymal white adipose tissue; iWAT, inguinal white adipose tissue.

To investigate the underlying mechanism how BAT-Exos worked, we tracked the *in vivo* distribution of BAT-Exos after intravenous injection, as the distribution is prerequisite for the function. Exosomes were stained with fluorescent dye DiR and injected into mice via intravenous injection. Then the distribution of exosomes was analyzed by *in vivo* imaging system. The results showed that after intravenous injection, most of the fluorescence-labeled exosomes accumulated in liver (Figure 6A-6C), which was consistent with previous reports. Notably, there were also abundant exosomes in the spleen and lung, though the abundance was much lower than that in the liver. The distribution profile suggests that liver might be at least one of the key target organs for the observed effects.

Next, to explore the exact effect of BAT-Exos on hepatocytes, cell metabolism analysis was performed using seahorse system. AML12 cells were seeded on a specialized plate and treated with PBS, Serum-Exos or BAT-Exos. The oxygen consumption rates (OCR) of cells with indicated treatments were monitored. The results showed robust increases of oxygen consumption in cells treated with BAT-Exos compared to cells treated with PBS or Serum-Exos, suggesting that BAT-Exos would boost energy expenditure in hepatocytes (Figure 6D).

### BAT-Exos function possibly via conferring components related with metabolism

**Figure 7:**
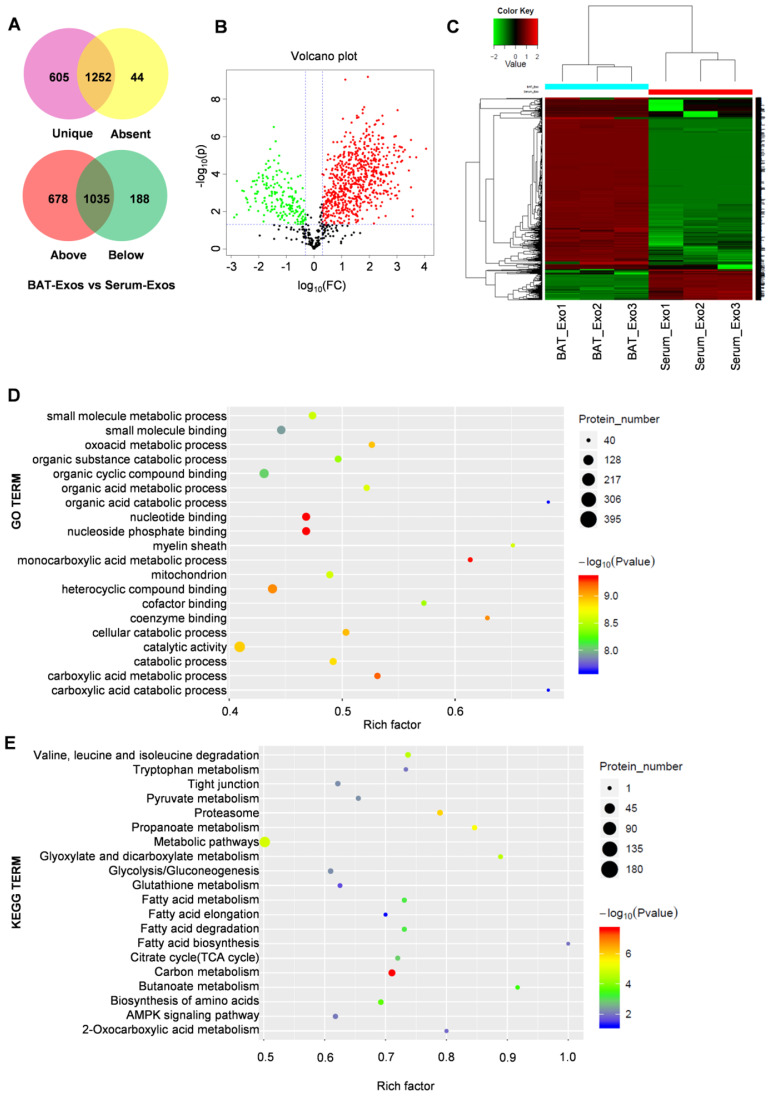
** Protein profiling of BAT-Exos by mass spectrometry. A-B.** Venn chart (A) and volcano plot (B) of differentially expressed proteins between BAT-Exos versus Serum-Exos. **C.** Cluster analysis of BAT-Exos and Serum-Exos. **D-E.** GO enrichment (D) and KEGG pathway enrichment analyses (E) of proteins up-expressed (Fold change ≥2, P < 0.05) in BAT-Exos. GO, gene ontology. Rich factor, proportion of differential proteins mapping to the present pathway relative to all identified proteins mapping to the pathway.

To figure out the molecular mechanism for the metabolic benefits of BAT-Exos, we next profiled the protein contents of BAT-Exos by mass spectrometry since proteins are the main bearers of life activities. A threshold of unique peptides>1, fold change ≥2, P<0.05 was used for screening of differential proteins. The results showed distinctive protein expression pattern of BAT-Exos and Serum-Exos (Figure 7A-C). A total of 1870 proteins were identified in BAT-Exos and 1301 in Serum-Exos. The expression levels of 678 proteins were higher in BAT-Exos compared to Serum-Exos and 188 were lower in BAT-Exos. Top 20 proteins up-expressed in BAT-Exos were shown in Table 1, most of which were components of mitochondria.

Enrichment analysis for differentially expressed proteins demonstrated significant enrichment in mitochondria components and extensive involvement in metabolic pathways of BAT-Exos (Figure 7D and 7E), which may account for the material bases of the metabolic effects of BAT-Exos. The gene set enrichment analysis (GSEA) for differential proteins showed similar results with proteins from BAT-Exos enriched in pathways including citrate cycle (TCA cycle), fatty acid degradation and oxidative phosphorylation (Figure S7).

To further confirm whether BAT-Exo delivery would cause increase of these proteins in the liver, we then examined the expression of Acadvl as an example in liver with control or BAT-Exos treatment. Acadvl is an enzyme also called very long-chain acyl-CoA dehydrogenase (VLCAD) and functions within mitochondria, which had both high abundance and fold change in the BAT-Exos, compared with Serum-Exos (Table 1). As expected, BAT-Exos treatment indeed increased the expression of Acadvl in the liver (Figure S8), indicating that BAT-Exos indeed transferred functional proteins into the recipient cells.

## Discussion

In this study, we proposed and verified the metabolic benefits of BAT-Exos from healthy young mice, which would be promising in treating obesity. The weekly treatment with BAT-Exos significantly reduced the body weights, lowered the blood glucose, and improved glucose tolerance of high-fat-diet fed mice. Abnormal cardiac function observed in HFD mice were also improved after BAT-Exos treatment. Consistently, lipid accumulation in the liver and adipose tissue were significantly reduced in BAT-Exos-treated HFD mice as compared to non-treated HFD mice. Cell metabolism analysis demonstrated that BAT-Exos could markedly boost energy expenditure in hepatocytes, which might account for the metabolic benefits observed in *in vivo* studies. Protein profiling by spectrometry revealed that BAT-Exos were rich in mitochondria components and were deeply involved in catabolism, providing material basis for their regulation of metabolism. Together, BAT-Exos might serve as a promising strategy for treating obesity and related disorders.

BAT features in its abundant number of mitochondria and high respiration rate and has been widely investigated as potential therapy for treating obesity and related metabolic disorders 27. Studies from different laboratories have demonstrated that transplantation of BAT could reverse obesity 28 and type 1 diabetes 29, regulate glucose homeostasis and insulin sensitivity 19 and ameliorate polycystic ovary syndrome (PCOS) 18. There have also been numerous studies exploring browning of white adipose tissue or enhancing activity of brown adipose tissue as anti-obesity strategies. Several molecules and pathways have been reported to be involved in white-to-brown adipose tissue conversion. To name a few, MCP-1 30, IEX-1 31, Notch signaling 32, FGF21 33, melatonin 34. However, the browning of adipose tissue was accompanied with adverse effects that would facilitate the metabolic dysfunction seen during hypermetabolic conditions 35. It is thus of great significance to utilize BAT in metabolic regulation in a controllable way.

Exosomes are nanovesicles that contain a wide variety of bioactive molecules including dsDNA, mRNA, miRNA, and proteins. Exosomes are secreted by almost all cells and can be taken up by cells adjacent or far away, influencing the physiological and biochemical status of the recipient cells. Previous studies have revealed that exosomes harbor the characteristics of their parental cells or tissues. For example, exosomes from stem cells are often reported to contribute to immunoregulatory effects and regeneration ability of the stem cells 36-38 and tumors exosomes have been proved to be involved in tumor invasion and metastasis 39-41 while exosomes from adipose tissue were reported to be responsible for obesity-triggered insulin resistance 42. Thomou *et al* reported that BAT-Exos might regulate FGF21 expression in the liver, suggesting potential effect of BAT-Exos on metabolism 43. As our understanding of exosomes progresses, more and more evidence showed that exosomes could reproduce key benefits of cells, making them attractive candidate for cell-free therapy 44-46. Compared to cell therapy, exosomes have several major advantages. As reviewed by Marban *et al* 25, contrary to fragile living cells, exosomes as tough lipid bilayer vesicles can go through repeated manipulation while remaining bioactive. Microvascular plugging or loss of transplanted cell viability which hinder the application of cell therapy are not problems for exosome therapy. Immune tolerability, high penetration across biological barriers and easy manipulation of exosomes also make them superior treatments than cells. Therefore, we hypothesized that exosomes from brown adipose tissue might share the metabolic regulation property of BAT and might be an ideal substitute of BAT in treating obesity. Though many studies have investigated potential application of BAT in treating obesity, most of the studies concerning BAT and obesity focused on BAT transplantation and browning of WAT, no researches regarding the direct effects of BAT exosomes on metabolism, especially in the context of obesity have been reported.

Numerous efforts have been put on exploring exosomes to treat metabolic disorders. For instance, Sun *et al* reported that MSC-derived exosomes alleviated type 2 diabetes by reversing peripheral insulin resistance and relieving β-Cell destruction 47. Exosomes from ADSC have also been reported to attenuate adipose inflammation and obesity through polarizing M2 macrophages and beiging of WAT 48. Ying et al reported that adipose tissue macrophage (ATM) exosomes from lean mice could improve glucose tolerance and insulin sensitivity with no significant changes in body weights when administered to obese recipients 49. In this study, we showed that BAT-Exos function directly by promoting energy expenditure, thus minimizing potential side effects, though the mechanisms of relieving β-Cell destruction, and improving insulin sensitivity could not be excluded.

In regard with the specific working mechanism of exosomes, there is still a lot of work to do. Although we have demonstrated that exosomes predominately accumulate in liver after intravenous injection which is consistent with other researches, there are still a small population of exosomes that will circulate into heart and WAT 50, 51. It is thus no doubt that some BAT-Exos could affect and regulate WAT and heart directly. In other words, the liver and thus remodeled metabolism might not be the sole mechanism of the therapeutic effects of BAT-Exos.

It is common to identify the key molecules encapsulated in the exosomes responsible for the biological function. However, the purpose to reveal the causal effect between a specific exosomal protein cargo and the functional change in the present study should be challenged. It is technically difficult to knock-in or knock-out a protein from the tissue derived exosomes. Moreover, there might be a theoretical protein functions more importantly than others. As shown in the proteomic data, there are abundant of proteins associated to metabolism, and might be functional when entering the recipient cells. In fact, we prefer the model that all the proteins play minor roles when function independently, however they would play a big role when work as a whole.

In reviewing the protein contents of BAT exosomes, we found that BAT-Exos also contain many other beneficial molecules, for example, eNAMPT which is reported to delay aging and extend lifespan. Meanwhile, BAT activity has been found to be inversely associated with aging 52, 53. Thus, whether BAT-Exos might have therapeutic effects on age-related diseases is worthy of investigation. In addition, clarification of the functional exosomal cargos in BAT-Exos also provides the clues for bioengineering of BAT-Exos from sources other than BAT.

Similar as most of the pioneering studies, there are some limitations concerning the present study. As to the purity of the exosomes, we used Exo-Quick for isolation of exosomes. Although the method was widely used to surely guarantee enough exosomes, it should be kept in mind that this method of exosome isolation cannot fully separate non-vesicular entities 54, which would be a problem when high purity is needed for studies like component decoding, cargo sorting, and signal transduction mechanisms. As to the cellular origin, the BAT-Exos should be originated from all the cell types in the brown adipose tissue, while mainly the mature brown adipocytes, which could be deduced from the proteomic data. Further clarification of the function of exosomes from the specific cell origin, especially the brown adipocytes, would be more informative. Currently, it remains challenging to obtain the specific exosomes either via isolation from cultured mature adipocytes or isolation exosomes with specific antibodies.

In summary, we have demonstrated in the present study with substantial evidence that BAT-Exos perform excellently in the fight against obesity, and thus could be considered as promising treatment for obesity. The results from cell metabolism analysis suggest that BAT-Exos could boost oxygen consumption. Ongoing studies decoding the detailed reactive molecules would further shed light on engineering of exosomes for the prevention and treatment of obesity and associated disorders.

## Materials and Methods

### Animal experiments

Healthy male C57BL/6J mice aged 8 weeks were purchased from the laboratory animal center of Fourth Military Medical University. All animal experiments were carried out in accordance with ethical regulations approved by the Animal Care and Use Committee of Fourth Military Medical University. Animals were randomly allocated to normal chow diet or high-fat diet (Research Diets, USA). Body weights of mice were monitored weekly. At the 8^th^ week, mice fed a high-fat-diet developed obesity (weighing 20% more than mice in NCD group). Two groups of HFD mice then started weekly treatment with 100 µg brown adipose tissue (BAT) exosomes or serum exosomes determined by protein concentration. The dosages of exosomes treatment vary from study to study 48, 49, 55. The 100 μg of exosomes per week was chosen based on our previous experience 56 and also in consideration of published literatures. After 6 weeks of exosome treatment, echocardiography was performed using Vevo 2100 imaging system (Visual Sonics, Canada) for evaluation of cardiac function of mice.

### Blood tests

For blood glucose test, mice were fasted at 8 a.m. for 6 hours before measurements as suggested by Andrikopoulos 57. For blood glucose tolerance test, mice were injected intraperitoneally with 2 mg/kg glucose and blood glucose were measured at 0 min, 15 min, 30 min, 60 min and 120 min after glucose administration. All experiments concerning blood glucose were performed via tail vein using glucometer ACCU-CHEK (Roche, Germany). For other bloods tests, blood samples were extracted from eyeballs of mice after anesthesia. 50-100 µl whole blood was stored in anticoagulant tube attached with EDTA for whole blood cell test, the rest of the blood was placed at room temperature for 20 min and then centrifugated at 3,000 ×g at 4 °C. After centrifugation, supernatants were harvested for further analyses. Indicators for serum testing include lipids contents such as total triglyceride (TG), total cholesterol (TC), liver function markers including alanine aminotransferase (ALT), aspartate aminotransferase (AST), cardiac function markers including creatine kinase (CK), L-lactic dehydrogenase (LDH-L) and creatine kinase- myocardial isoenzymes (CK-MB). Blood biochemical assays were done by corresponding assay kits (HUILI, China) and blood cell analyses were performed on automated hematology analyzer BC-2800vet (Mindray, China).

### Exosome isolation and characterization

For BAT exosome isolation, male C57BL/6 mice aged 6 to 8 weeks were used and brown adipose tissue were obtained from their interscapular region under sterile condition. Brown adipose tissues were cut into extremely small pieces and were cultured in serum-free DMEM for 24 hours. Supernatants of the cultured BAT were collected and centrifugated at 3,000 × g for 15 min for removal of tissue debris. The fluids were then filtered through 0.22 µm filter and the filtrate were precipitated with ExoQuick-TC overnight at 4 °C. Then the mixture was centrifugated at 4 °C, 1500×g for 30 min. Supernatants were discarded and precipitated exosomes were concentrated by another round of centrifugation at 1500×g, 10 min. The remaining fluids were aspirated and the obtained pellets were resuspended in PBS at an appropriate concentration. For isolation of serum exosomes, mice were anaesthetized and then their eyeballs were excised for collection of blood. The blood was placed at room temperature for 20 min and then centrifugated at 4 °C, 2000 × g for 15 min. The supernatants were carefully aspirated and subjected to same protocols for exosome isolation as described above. For characterization of exosomes, particle size analysis was performed using NanoPlus (Otsuka Electronics, Japan). Morphology of exosomes was observed under transmission electron microscopy (JEOL, Japan), and western blot was performed for protein content analysis of exosomes. Specific procedures were the same as previously described 56. Primary antibodies used in the study included anti-GM130 (sc71166, Santa Cruz, USA), anti-TSG101 (ab83, Abcam, USA), anti-CD81 (ab109201, Abcam, USA), and anti-ACADVL (ab155138, Abcam, USA). Second antibodies used in the study included HRP-conjugated goat anti-rabbit IgG (D110058, BBI, China) and HRP-conjugated goat anti-mouse IgG (D110087, BBI, China).

### Animal echocardiography

Animal echocardiography was performed by experienced technicians using Vevo 2100 Imaging System (FUJIFILM VisualSonics, Canada) and each parameter was repeatedly measured for six times. Mice were anesthetized by isoflurane (RDW, China) on a heating pad and had their hair removed around their chest and abdomen. The heartbeat of mice was kept between 400-500 beats per minute during the examination. Parasternal long axis view (PLAX), short axis view and four-chamber view were scanned for multiple cardiac functional parameters.

### *In vivo* fluorescence imaging

Exosomes were labeled with fluorescent dye DiR and injected into HFD mice through tail vein for exosome distribution analysis. Four hours later, the distribution of fluorescent-labeled exosomes was observed under using *in vivo* imaging system (IVIS, PerkinElmer, USA). Mice were anesthetized by isoflurane and had their thoracic and abdominal hair removed before imaging. After whole-body imaging, mice were sacrificed and their organs were taken out for subsequent fluorescence imaging.

### Cell metabolism analysis

Oxygen consumption rate (OCR) of cells was tested using Seahorse XFe24 Metabolism Analyzer (Agilent, USA). AML12 cells were seeded on XF24 cell culture plates at a density of 1×10^-5^ cells per well 24 hours prior to detection and were treated with BAT-Exos or Serum-Exos 6 hours after the seeding. The dosage of exosome treatment was based on previous reports 48, 49, 55. Before subjected to analyzer, cells were washed with Seahorse XF Cell Energy Phenotype Test Assay Medium and placed in a 37 °C incubator without CO_2_ for one hour. For mitochondrial stress test, cells were sequentially injected with Oligomycin, FCCP, and Rotenone + antimycin A to assess different components of oxygen consumption.

### Protein profiling

Exosomes isolated from BAT or serum were resuspended in PBS and shipped to authorize institution at low temperatures in dry ice for mass spectrometry analysis. Exosomes isolated from 3 mice were assigned as one sample and each group contained 3 samples. The protein profiling by mass spectrometry and subsequent bioinformatics analyses were done by BangFei Bioscience (Beijing, China). Briefly, total protein of BAT-Exos was extracted with RIPA lysis buffer and electrophoresis was done to grossly asses protein quality. Then, sample proteins were digested by trypsin and obtained products were separated by high performance liquid chromatography (HPLC) and then analyzed by Orbitrap Fusion Lumos (Thermo Scientific, USA). A threshold of unique peptides>1, fold change ≥2, P<0.05 was used for screening of differential proteins. Differentially expressed proteins were analyzed by gene ontology (GO) enrichment, KEGG pathway enrichment, as well as gene set enrichment analysis (GESA).

### Statistical analysis

Data are presented as mean ± SEM. Student t tests was used for two group comparison and ANOVA was used for comparison of differences between groups while multiple comparison were performed with post hoc analysis (GraphPad Prism 7.0). Statistical significance was defined as P < 0.05.

## Supplementary Material

Supplementary methods, figures and tables.

## Figures and Tables

**Figure 8:**
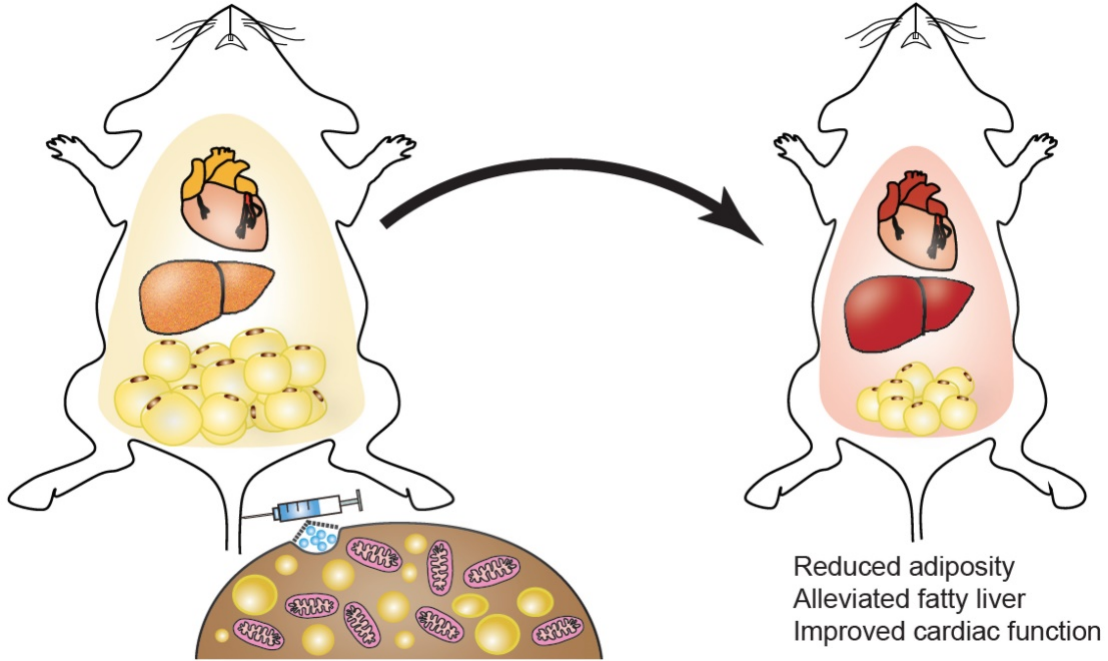
** Schematic summary of the study.** Intravenous injection of exosomes isolated from brown adipose tissue can lead to reduced adiposity, alleviated fatty liver, improved cardiac function, thus mitigating metabolic syndrome in HFD mice.

**Table 1:** Top 20 proteins most up-regulated in BAT-Exos

Accession	Gene name	Unique peptides	Fold change	P value
Q9D2G2	Dlst	9	11415.36246	4.50E-06
P13707	Gpd1	24	5015.498011	1.28E-05
Q91ZJ5	Ugp2	22	3758.13794	0.018092505
Q921L4	LOC665622	7	3713.0007	0.008470212
P17563	Selenbp1	22	3241.106903	1.52E-06
P49817	Cav1	8	3226.903646	0.000525067
Q3UAG2	Pgd	22	2947.502253	1.23E-05
Q9EQ20	Aldh6a1	21	2720.734984	6.52E-05
Q8BWT1	Acaa2	20	1933.65099	5.11E-06
Q99LC5	Etfa	14	1692.953789	6.47E-06
P09411	Pgk1	18	1551.853574	1.93E-05
Q9CZU6	Cs	11	1515.512174	3.77E-06
Q76MZ3	Ppp2r1a	20	1418.840383	2.20E-05
P50544	Acadvl	31	1378.92344	3.64E-06
Q9D8N0	Eef1g	20	1251.109562	3.47E-06
Q3TIC8	Uqcrc1	12	1220.424648	1.12E-05
Q9DB77	Uqcrc2	13	1207.877488	0.000840767
Q3U1J4	Ddb1	32	1153.403278	1.27E-05
P97807	Fh	15	1142.379708	5.77E-06
Q5SX40	Myh1	22	1101.99462	0.000387361
