# Supplementary material for: Brown adipose tissue-derived exosomes mitigate the metabolic syndrome in high fat diet mice
Source: Theranostics. 2020 Jul 9;10(18):8197–210. doi: 10.7150/thno.43968 (PMC7381731; doi:10.7150/thno.43968)
Supplement: Supplementary file 1 — Supplementary methods, figures and tables. [file thnov10p8197s1.pdf]

Supplementary information

Brown adipose tissue-derived exosomes mitigate the metabolic syndrome in high fat diet mice

Xueying Zhou[1,2,#], Zhelong Li[1,2,#], Meihao Qi[3,#], Ping Zhao[1], Yunyou Duan[1], Guodong Yang[2,*], Lijun Yuan[1,*]

[1] Department of Ultrasound Diagnostics, Tangdu Hospital, Fourth Military Medical University, Xi'an, People's Republic of China

[2] The State Laboratory of Cancer Biology, Department of Biochemistry and Molecular Biology, Fourth Military Medical University, Xi'an, People's Republic of China

[3] Department of Otolaryngology Head and Neck Surgery, Xijing Hospital, Fourth Military Medical University, Xi'an, People's Republic of China

# These authors contributed equally to this article.

* Correspondence authors:

Lijun Yuan, Department of Ultrasound Diagnostics, Tangdu Hospital, Fourth Military Medical University, Xinshi Road NO.569th, 710038, Xi'an, China, email: yuanlj@fmmu.edu.cn ; Tel: +862984777471, Fax: +862984777471.

Guodong Yang, The State Laboratory of Cancer Biology, Department of Biochemistry and Molecular Biology, Fourth Military Medical University, Changlexi Road NO.169th, 710032, Xi'an, China, email: yanggd@fmmu.edu.cn; Tel: +862984774516, Fax: +862984774516.

Methods

Realtime quantitative PCR

Total RNA of indicated tissues was extracted using Trizol Reagent (Invitrogen, USA) following manufacturer's instruction. The purity and concentration of isolated RNA were determined by NanoDrop 2000 spectrophotometer (Thermo, USA). Reverse-transcription was conducted using PrimeScript^{TM} RT Master Mix (Takara, Japan) for synthesis of cDNA. Realtime quantitative PCR was performed using FastStart Essential DNA Green Master (Roche). β-actin was used as reference gene. Relative expression was calculated based on the 2^{-ddCt} method. The sequences of PCR primers were provided in Table S2.

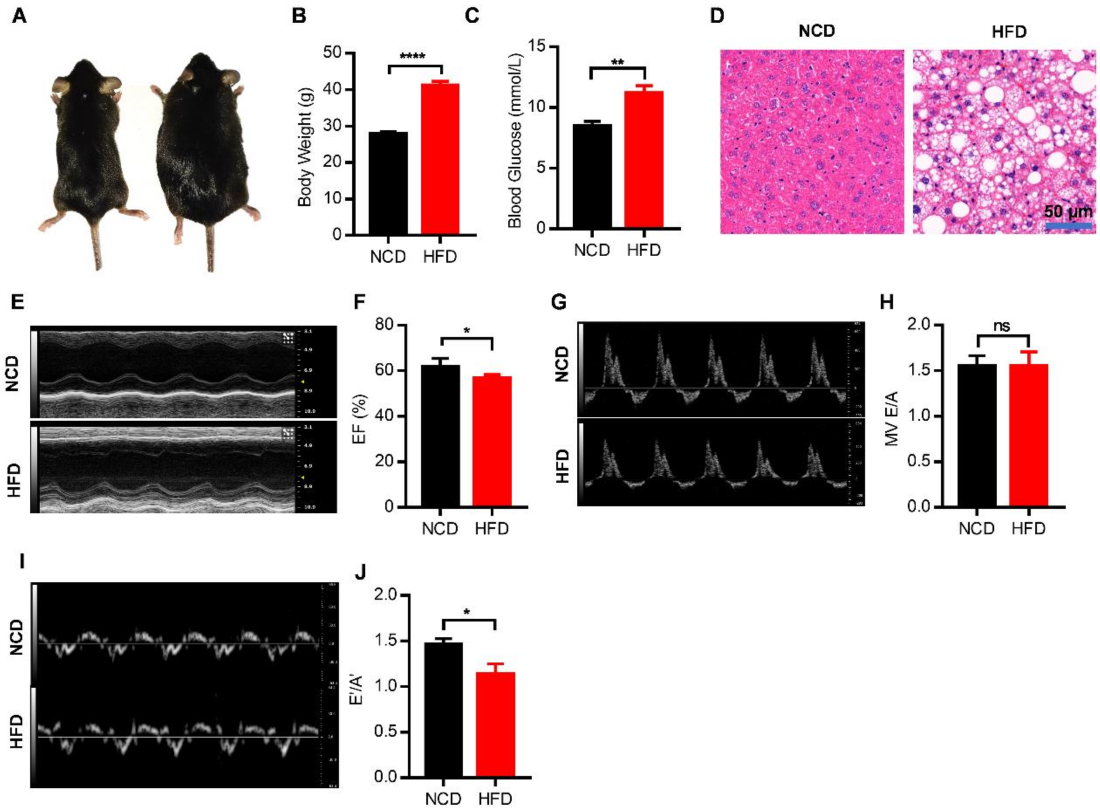

Figure S1 Obese mice induced by high-fat-diet feeding exhibit metabolic disorder and cardiac dysfunction. A. Representative images of NCD mice and HFD mice. B-C. Body weight (B) and blood glucose (C) of indicated mice. D. HE staining of liver section of NCD mice and HFD mice. E-J. Representative images of echocardiography evaluation of indicated mice and quantification of cardiac functional parameters. Data are presented as mean±SEM. n=5 per group, $^{*}P < 0.05$, $^{**}P < 0.01$, $^{****}P < 0.0001$. Scale bar=50 μm. NCD, normal chow diet; HFD, high-fat diet. EF, ejection fraction.

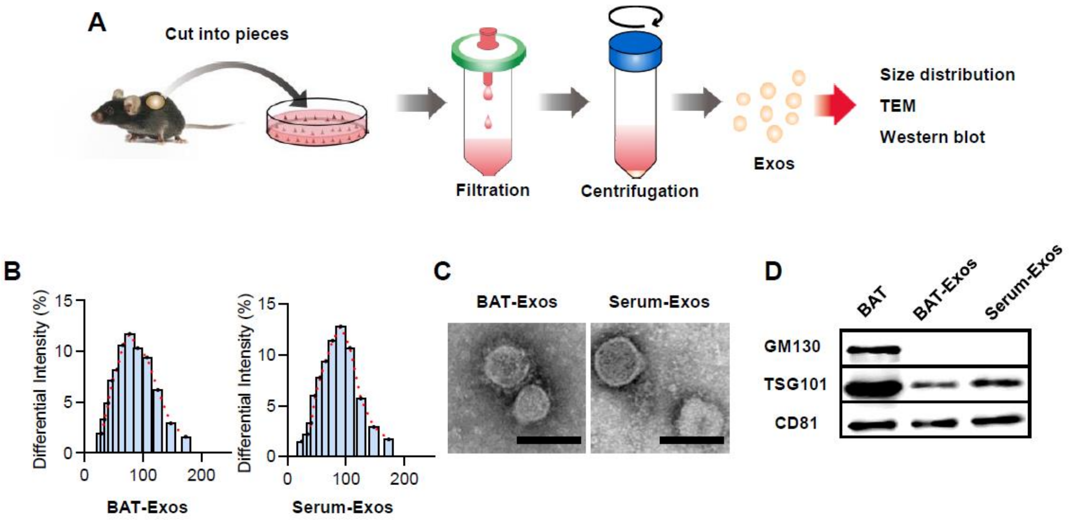

Figure S2 Isolation and identification of BAT-derived exosomes. A. Illustration of the procedure of BAT exosome isolation. B. Size distribution of exosomes derived from BAT or serum. C. Representative images of exosomes observed under transmission electron microscope. Scale bar=100 μm. D. Western blot analysis of BAT and isolated exosomes from BAT and Serum.

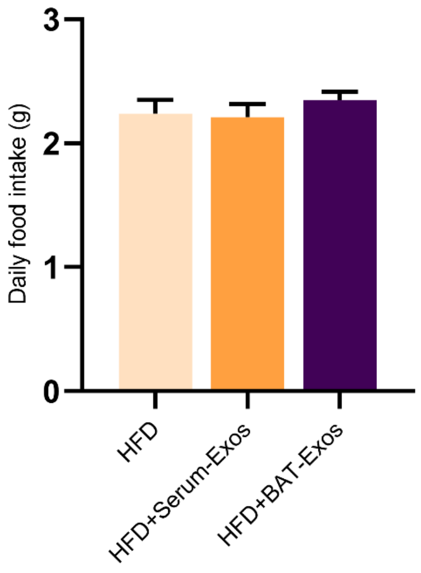

Figure S3 Effects of exosome treatment on daily food intake. Data are presented as mean±SEM. n=6 per group.

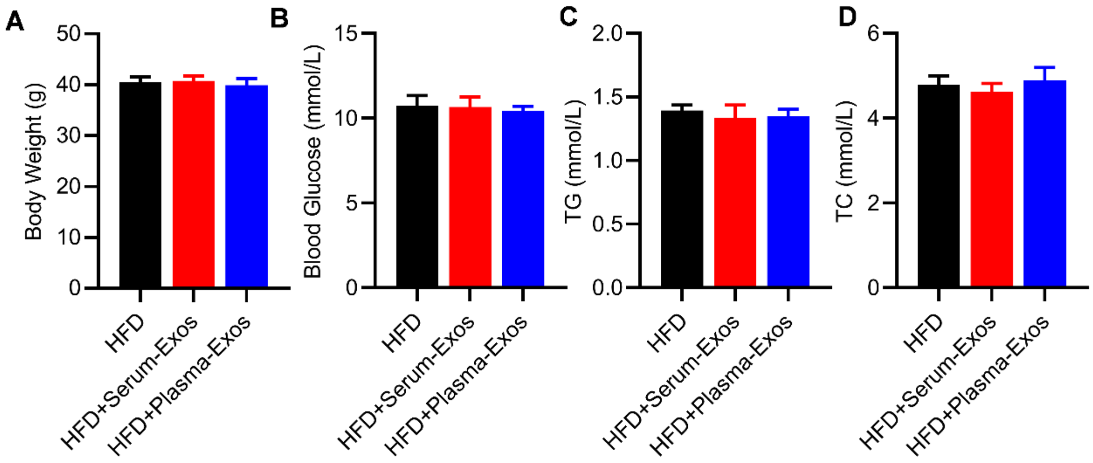

Figure S4 Comparison of the metabolic effects of serum and plasma exosomes on HFD mice. A-D. Mice fed with high fat diet for 14 weeks were additionally treated with either serum exosomes or plasma exosomes in the last 6 weeks. Body weight (A), blood glucose (B), TG (C) and TC (D) of mice in each group were measured. HFD, high fat diet; TG, triglyceride; TC, total cholesterol.

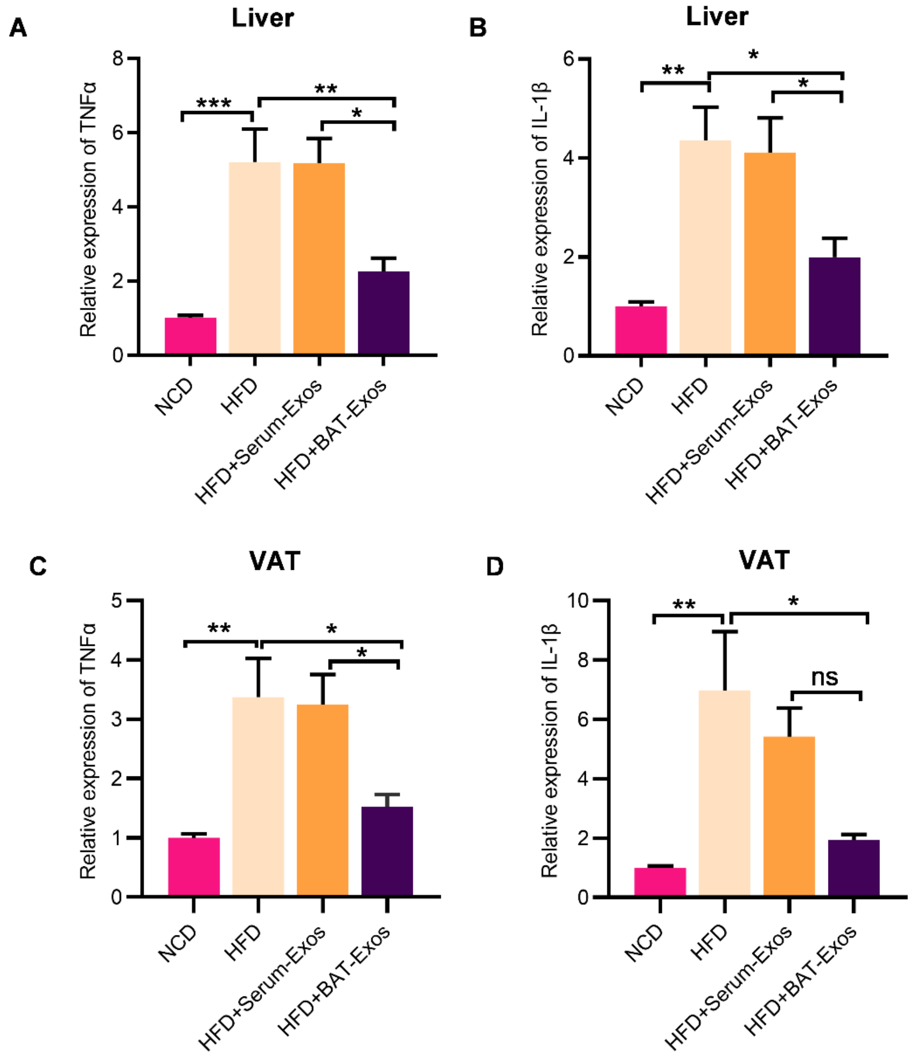

Figure S5 Expression of inflammatory cytokines in the liver and visceral adipose tissue. A-B. Expression of TNFα (A) and IL-1β (B) in the liver of indicated mice. C-D. Expression of TNFα (C) and IL-1β (D) in the visceral adipose tissue of indicated mice. Data are presented as mean±SEM. n=6 per group, $^{**}P < 0.05$, $^{**}P < 0.01$, $^{***}P < 0.001$. VAT, visceral adipose tissue.

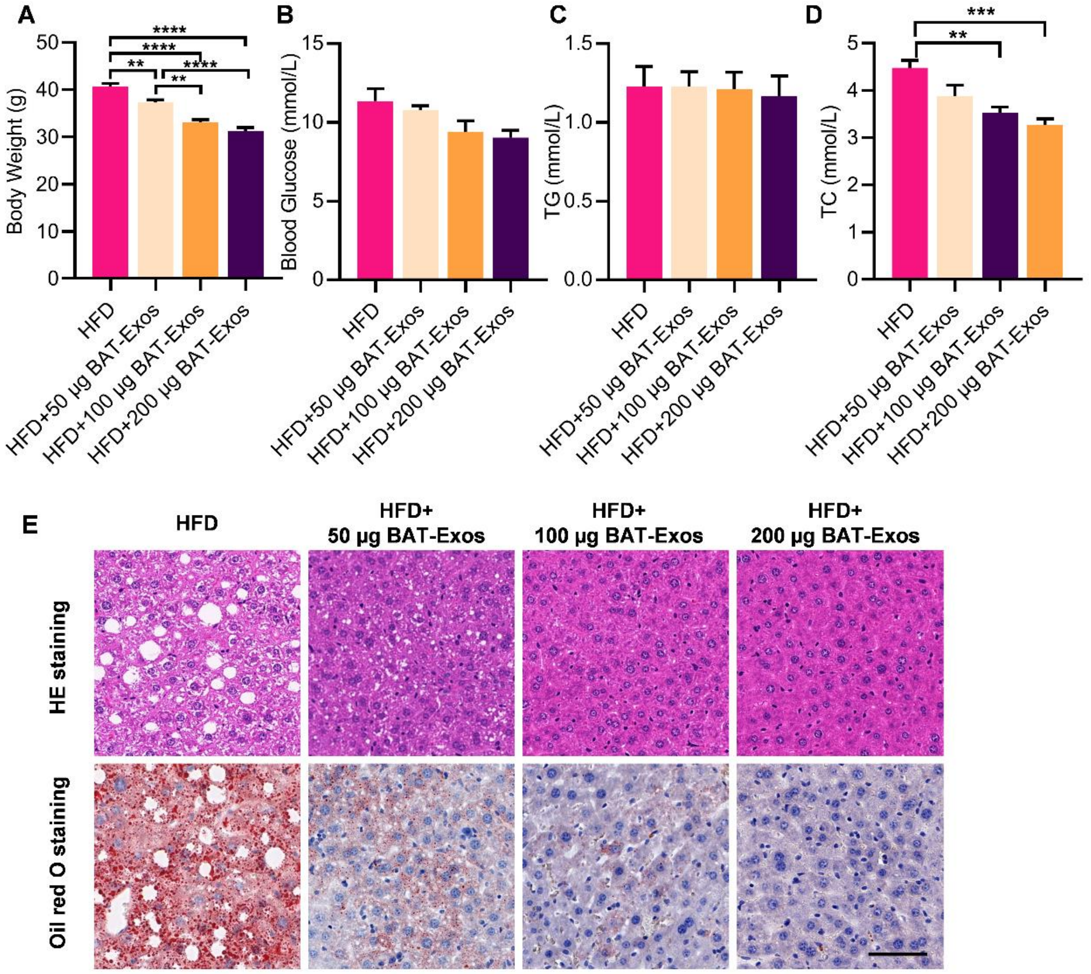

Figure S6 BAT-Exos mitigate metabolic disorder in HFD mice in a dose-dependent way. A-D. Mice fed with high fat diet for 14 weeks were additionally treated with different doses of BAT-Exos in the last 6 weeks. Body weight (A), blood glucose (B), TG (C) and TC (D) of mice in each group. E. HE staining and Oil red o staining of liver sections of mice in each group. Data are presented as mean±SEM. n=5 per group. HE, hematoxylin-eosin; HFD, high fat diet; TG, triglyceride; TC, total cholesterol. Scale bar=50 μm. $^{**}P < 0.01$, $^{***}P < 0.001$, $^{****}P < 0.0001$.

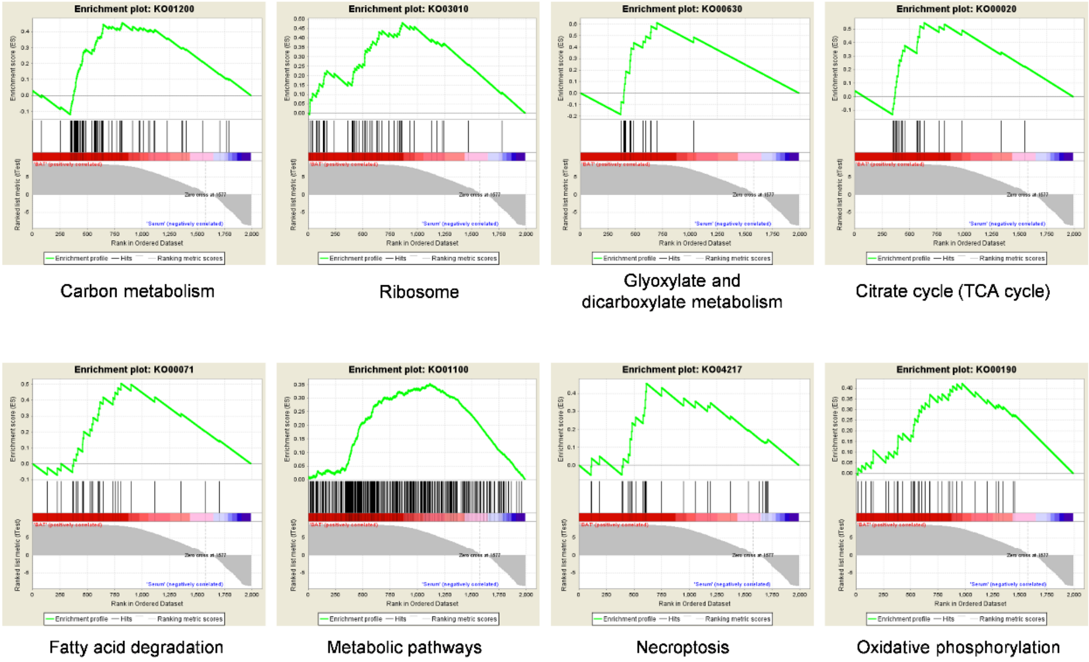

Figure S7 Gene set enrichment analysis (GSEA) of proteins up-expressed (Fold change ≥2, P < 0.05) in BAT-Exos. Shown are top 8 KEGG pathways enriched in BAT-Exos.

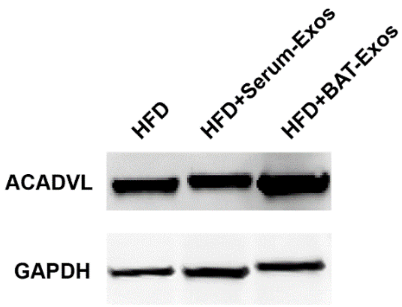

Figure S8 Western blot analysis of ACADVL in the liver. Mice were injected with 200 μg Serum-Exos or BAT-Exos, and the liver were harvested 24 h post exosome injection for western blot assay. GAPDH served as internal control. Representative data of 3 independent experiments.

Table S1 Results of blood cell tests

Parameters	Unit	NCD	HFD	HFD+Serum-Exos	HFD+BAT-Exos
WBC	10^9/L	4.60±0.17	4.22±0.20	4.57±0.15	4.50±0.26
Lymph	10^9/L	3.68±0.13	3.52±0.18	3.82±0.14	3.67±0.14
Mon	10^9/L	0.12±0.01	0.08±0.01	0.08±0.01	0.13±0.02
Gran	10^9/L	0.80±0.04	0.62±0.02	0.67±0.03	0.65±0.09
Lymph%	%	80.27±0.53	82.42±0.51	83.22±0.74	82.10±1.49
Mon%	%	3.23±0.06	2.63±0.13	2.53±0.12	2.70±0.23
Gran%	%	16.50±0.48	14.95±0.44	14.25±0.65	15.12±1.42
RBC	10^{12}/L	5.36±0.11	5.72±0.16	5.95±0.15	5.94±0.06
HGB	g/l	74.83±1.07	80.33±1.75	81.33±1.67	81.17±1.77
HCT	%	27.18±0.15	28.63±0.64	28.53±0.65	27.23±0.47
MVC	fl	51.35±0.84	50.50±0.70	48.32±0.71	46. 37±0.84
MCH	pg	13.98±0.13	14.08±0.19	13.70±0.22	13.80±0.35
MCHC	g/l	274.67±3.32	280.50±2.71	285.17±2.30	297.83±4.45
RDW	%	22.87±0.25	22.45±0.10	22.70±0.23	23.53±0.30
PLT	10^9/L	858.67±21.06	765.00±24.12	813.17±26.35	925.67±20.52
MPV	fl	4.92±0.09	4.73±0.05	4.80±0.05	4.63±0.04
PDW		16.88±0.09	16.75±0.08	16.52±0.09	16.75±0.13
PCT	%	0.42±0.01	0.36±0.01	0.39±0.01	0.42±0.01

WBC, white blood cell; Lymph, lymphocyte; Mon, monocyte; Gran, granulocyte; RBC, red blood cell; HGB, hemoglobin; HCT, hematocrit; MVC, mean corpuscular volume; MCH, mean corpuscular hemoglobin; MCHC, mean corpuscular hemoglobin concentration; RDW, RBC distribution width; PLT, Platelet; MPV, mean platelet volume; PDW, platelet distribution width; PCT, platelet crit. Data are presented as mean±SEM. n=6 per group.

Table S2 Sequences of qPCR primers used in the study.

**Primer name**	**Sequences**
β-actin forward	5'-CTGTCCCTGTATGCCTCTG-3'
β-actin reverse	5'-ATGTCACGCACGATTTCC-3'
TNFα forward	5'-GCAACTGTTCCTGAACTCAACT-3'
TNFα reverse	5'-ATCTTTTGGGGTCCGTCAACT-3'
IL-1β forward	5'-CCCTCACACTCAGATCATCTTCT-3'
IL-1β reverse	5'-GCTACGACGTGGGCTACAG-3'
